# NLRP3 Inflammasome—A Key Player in Antiviral Responses

**DOI:** 10.3389/fimmu.2020.00211

**Published:** 2020-02-18

**Authors:** Chunyuan Zhao, Wei Zhao

**Affiliations:** ^1^Key Laboratory of Infection and Immunity of Shandong Province, Department of Immunology, School of Basic Medical Science, Shandong University, Jinan, China; ^2^State Key Laboratory of Microbial Technology, Shandong University, Jinan, China; ^3^Department of Cell Biology, School of Basic Medical Science, Shandong University, Jinan, China

**Keywords:** NLRP3, inflammasome, antiviral immunity, viral infection, viral evasion

## Abstract

The NACHT, LRR, and PYD domains-containing protein 3 (NLRP3) inflammasome is an oligomeric complex comprised of the NOD-like receptor NLRP3, the adaptor ASC, and caspase-1. This complex is crucial to the host's defense against microbes as it promotes IL-1β and IL-18 secretion and induces pyroptosis. NLRP3 recognizes variety of pathogen-associated molecular patterns (PAMPs) and danger-associated molecular patterns (DAMPs) generated during viral replication that triggers the NLRP3 inflammasome-dependent antiviral immune responses and facilitates viral eradication. Meanwhile, several viruses have evolved elaborate strategies to evade the immune system by targeting the NLRP3 inflammasome. In this review, we will focus on the crosstalk between the NLRP3 inflammasome and viruses, provide an overview of viral infection-induced NLRP3 inflammasome activation, and the immune escape strategies of viruses through their modulation of the NLRP3 inflammasome activity.

## Introduction

A variety of pathogenic viruses can cause severe diseases and are threats to human health, such as hepatitis C virus (HCV), human immunodeficiency virus-1 (HIV-1), influenza A virus (IAV), and Zika virus (ZIKV). To eradicate invading viruses quickly and efficiently, host has evolved highly conserved sensors, called pattern recognition receptors (PRRs) (1), that recognize viral infections and, subsequently, trigger antiviral immune responses. PRRs, which include Toll-like receptors (TLRs), retinoic acid-inducible gene-I (RIG-I) like receptors (RLRs), and DNA sensors such as cyclic GMP-AMP synthase (cGAS), sense different pathogen-associated molecular patterns (PAMPs), and damage-associated molecular patterns (DAMPs) derived from invading viruses. Upon engagement with their cognate ligands, PRRs can induce the activation of two different transcription factor-mediated pathways, IRF3 and NF-κB. IRF3 mediates the secretion of type I interferons (IFNs), which lead to the activation of the JAK-STAT pathway and the expression of interferon-stimulated genes (ISGs) (2). NF-κB initiates both the production of proinflammatory factors, such as tumor necrosis factor (TNF)-α and interleukin (IL)-6, as well as the initiation of inflammasome priming stage (see below).

Some PRRs, such as NACHT, LRR, and PYD domains-containing protein 1 (NLRP1), NLRP3, NLR family CARD domain-containing protein 4 (NLRC4), and absent in melanoma 2 (AIM2), recruit apoptosis-associated speck-like protein (ASC) and caspase-1 to form the inflammasome—a multimeric platform of proteins that initiates inflammation as well as some forms of cell death (3). Among all the inflammasomes discovered, the NLRP3 inflammasome is the most extensively studied and it plays an important role in both inflammation and antiviral responses. However, the mechanisms of the NLRP3 inflammasome activation are still complicated and remain controversial. In this review, we will focus on the recent research advances made in terms of NLRP3 inflammasome activation during a viral infection and the immune evasion mechanisms of viruses that target the NLRP3 inflammasome.

## The Activation Of The NLRP3 Inflammasome

The roles of the NLRP3 inflammasome are vital in the host antiviral immune responses. Several viruses, such as IAV and West Nile virus (WNV), tend to induce an appropriate and early phase activation of the NLRP3 inflammasome. As a result, activation of the NLRP3 inflammasome inhibits viral replication and reduces mortality in mouse models (4, 5). The NLRP3 inflammasome can be activated by sensing viral components as well as cytosolic danger signals, such as mitochondria injury, protein aggregates, and aberrant ion concentrations, all of which can be caused by a viral infection.

**Figure 1:**
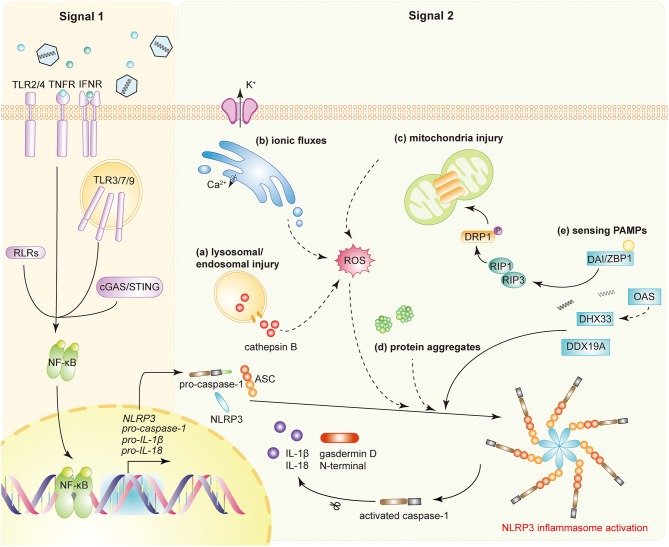
NLRP3 inflammasome activation during viral infections. Activation of the NLRP3 inflammasome requires two signals. Signal 1 (priming signal): the activation of PRRs, TNFR, or IFNR induces NF-κB activation, triggers the transcription of NLRP3, pro-caspase-1, pro-IL-1β, and pro-IL-18. Signal 2 (activation signal): multiple DAMPs and PAMPs induce NLRP3 inflammasome assembly and activation. DAMPs include **(a)** lysosomal or endosomal injury, **(b)** aberrant ionic fluxes, **(c)** mitochondrial injury, and **(d)** protein aggregates. **(e)** With the help of DAI/ZBP1, DHX33, OAS, or DDX19A, NLRP3 is activated by sensing viral proteins and RNA. NLRP3 inflammasome activation leads to the auto-cleavage of pro-caspase-1. Caspase-1 then mediates the proteolytic process of pro-IL-1β, pro-IL-18, and gasdermin D (GSDMD).

NRLP3 inflammasome activation requires two steps (Figure 1). The first step, known as the priming step, is induced by PRRs or TNFR activation. This leads to the activation of NF-κB and promotes the expression of NLRP3, pro-IL-1β, and pro-IL-18. Additionally, IFNAR also activates the priming stage of NLRP3 inflammasome activation (6). The second step, also called the activation step, is triggered by a range of stimuli that emerge during infections, tissue damage, or metabolic imbalances. Such stimuli include ATP, pore-forming toxins, crystalline substances, nucleic acids, and invading pathogens (7). NLRP3 recruits ASC through its N-terminal pyrin domain (PYD) by homophilic interactions, resulting in the formation of ASC prion-like oligomerizes (8). The NAIP, CIITA, HET-E and TP1 (NACHT) domain in the middle of the NLRP3 possesses dNTPase activity and mediates downstream oligomerization (9). The C-terminal leucine-rich repeat domain (LRR) associates with HSP90, SGT1, and PML and is considered to be responsible for the regulation of NLRP3 inflammasome activity (10, 11).

Once assembled, the NLRP3 inflammasome triggers the auto-cleavage of pro-caspase-1 (12, 13). As an effect factor, caspase-1 mediates the proteolytic processing of pro-IL-1β, pro-IL-18, and the propyroptotic factor gasdermin D (GSDMD) (14). GSDMD forms pores in the membrane of infected cells, facilitating the secretion of IL-1β/IL-18 and inducing the inflammation-associated cell death known as pyroptosis (15). The secretion of IL-1β subsequently recruits neutrophils to the inflammatory site to aid in the elimination of invading viruses (16). Moreover, both IL-1β and IL-18 are responsible for the subsequent induction of the adaptive immune response (17, 18). Accordingly, optimal activation of the NLRP3 inflammasome facilitates the establishment of a host antiviral status.

However, aberrant NLRP3 inflammasome activation can also lead to severe pathological injury. In an IAV infection model, juvenile mice had sustained elevated levels of type I IFNs and persistent NLRP3 inflammasome activation, suffering from severe lung injury independent of viral titer (19). In addition, HIV-1 infected microglia are shown to cause NLRP3-associated neuroinflammation (20). HCV infection promotes chronic intrahepatic inflammation and liver injury mediated by the NLRP3 inflammasome (21). The recruitment of excessive inflammatory cells and pyroptosis-mediated cell damage take part in the immunopathology progresses (22, 23).

## Priming Step Of NLRP3 Inflammasome Activation In Viral Infection

In the rest state, cellular NLRP3 level is low enough to avoid aberrant inflammasome assembly and activation. Viral infection initiates NF-κB signaling through the activation of PRRs-dependent pathways (24, 25). RLRs, as cytosolic RNA sensors, detect viral RNA, such as VSV and IAV (26, 27). TLR3, TLR7, and TLR9 participate in the sensing of IAV, HCV, and adenovirus type 5 (Ad5) (21, 27, 28). Respiratory syncytial virus (RSV), IAV and human parainfluenza virus (HPIV) activate TLR2 or TLR4 in macrophages (29–31). Herpes simplex virus type 1 (HSV-1) infection could be detected by cGAS, a major cytosolic DNA sensor (32). Research indicates that HIV can prime NLRP3 inflammasome transcription in monocyte-derived macrophages (33). Moreover, PRRs induced IFN-β and TNF-α that could, in turn, activate NF-κB and provide the cascade amplification necessary for NLRP3 inflammasome activation. This response enables the host to defend effectively against viral infections.

## Viral Infection Triggered The Activation Step Of NLRP3 Inflammasome

The NLRP3 inflammasome can be activated by both viral components, including RNA and proteins (PAMPs), and danger signals (DAMPs). Although it does not directly interact with viral structures, the NLRP3 inflammasome is still sensitive to invading viruses and cytosolic danger signals, indicating its complicated mechanisms of sensing invading pathogens.

## PAMPs

NLRP3 can sense some PAMPs with the help of other receptors. In primary human monocyte-derived macrophages, the DExD/H-box RNA helicase family member, DHX33, senses reoviral genomic RNA, interacts with NLRP3 to form the inflammasome complex, and leads to the secretion of IL-1β (34). 2′,5′-oligoadenylate (2–5A) synthetase (OAS) recognizes dsRNA from some viruses, such as IAV and VSV, and promotes the cleavage thereof by endoribonuclease RNase L; the cleaved nucleic acids then be detected by DHX33 (35). Furthermore, DDX19A, another DExD/H-box RNA helicase family member, senses porcine reproductive and respiration syndrome virus (PRRSV) and promotes NLRP3 inflammasome activation (36).

With the help of DAI/ZBP1, NLRP3 recognizes viral proteins and promotes inflammasome assembly (37). DAI/ZBP1 interacts with the IAV nucleoprotein (NP) and polymerase subunit PB1 after infection. DAI/ZBP1 subsequently interacts with RIP3 through their shared domain homotypic interaction motif (RHIM), to activate the NLRP3 inflammasome via the RIP1-RIP3-caspase-8 pathway (37–39). It is evident that the viral protein sensor DAI/ZBP1 is critical to the induction of NLRP3 inflammasome-mediated apoptotic and necroptotic cell death since DAI/ZBP1 deficient mouse were protected from mortality during IAV infection. However, viral RNA or proteins alone were not sufficient to induce DAI/ZBP1-mediated cell death during IAV infection (40). Instead, during viral replication, DAI/ZBP1 senses the viral ribonucleoprotein (vRNP), containing IAV RNA, NP, and PB1, and subsequently initiates programmed cell death (40).

## DAMPs

To date, no ligand that binds directly to NLRP3 has been found. Accordingly, the NLRP3 inflammasome is usually associated with sensing cytosolic danger signals referred to as DAMPs. Not only intact viruses, such as IAV, SeV, HSV, and adenovirus (AdV), but also viral components, including internalized or genomic DNA, dsRNA, ssRNA, and even poly(I:C), could directly activate the NLRP3 inflammasome and induce IL-1β secretion in macrophages (5, 28, 41–43). During infection, viruses cause a series of changes in cellular status of their host cells, including lysosomal maturation, aberrant ion concentrations, mitochondria damage, and the accumulation of misfolded protein aggregates, all of which are recognized as danger signals by the host and lead to the activation of the NLRP3 inflammasome.

The maturation and acidification of lysosomes lead to the leaking of catalytically active cathepsin B, and the subsequent generation of reactive oxygen species (ROS), which, in turn, activates the NLRP3 inflammasome (5, 44). AdV type 5 induces the disruption of endosomal membranes and the release of cathepsin B, thereby activating NLRP3 (28, 45). This activation is required for the lysosomal localization and membrane penetration ability of AdV, since the temperature-sensitive mutant of Ad5 cannot induce the activation of NLRP3. ROS are also required for NLRP3 inflammasome activation since lower levels of IL-1β were observed in the presence of NADPH oxidase inhibitors or the oxygen scavenger N-acetylcysteine. IAV infection, or RNA species, activates the NLRP3 inflammasome by inducing lysosomal acidification (5).

An appropriate ionic concentration is crucial to maintain cellular homeostasis within host cells. However, once homeostasis is disrupted, the NLRP3 inflammasome will sense danger signals and activate accordingly. Potassium efflux is a well-known activator of the NLRP3 inflammasome (46, 47). HCV infection induces potassium efflux in macrophages, thus leading to the maturation of pro-IL-1β (21).

Viroporins are small, highly hydrophobic proteins derived from viruses, which interact with membranes to modify the host cell's permeability to ions or other small molecules (48). Several viroporins are observed to localize to the Golgi apparatus and other cytoplasmic structures during viral infection (49–51). Examples include 2B proteins from EMCV, poliovirus, enterovirus 71 (EV71), and human rhinoviruses (HRV), the envelope (E) protein of severe acute respiratory syndrome coronavirus (SARS-CoV), as well as influenza virus M2 protein. These viroporins activate the NLRP3 inflammasome by inducing different ionic fluxes. Other viral proteins, such as non-structural 2B proteins from EMCV, HRV, poliovirus and EV71, as well as N protein from SARS-CoV, cause the flux of calcium from intracellular storages to the cytosol, which is indispensable for NLRP3 activation (49–51). The HCV core protein regulates intracellular calcium flux through a phospholipase C-dependent process, instead of directly changing the membrane permeability (52). HRV infection also induces the co-localization of NLRP3 and NLRC5, which sense calcium fluxes and assemble in a cooperative manner (50). Influenza virus M2, RSV small hydrophobic (SH) protein, and SARS-CoV viroporin 3a change membrane permeability by forming a cation-selective ion channel. As a result, the ion channel permits the release of Na^+^/K^+^, rather than Ca^2+^, to induce the NLRP3 inflammasome activation (53–55). The disturbance of ionic concentrations leads to mitochondria damage and the production of ROS, potentiating NLRP3 inflammasome activation (55).

Mitochondria damage is also a crucial activator of the NLRP3 inflammasome. Similar to lysosomal or endosomal maturation, mitochondria damage also induces the production of ROS to activate the NLRP3 inflammasome (56, 57). The RIP1-RIP3 complex, assembled after viral infection, induces activation of the GTPase DRP1. DRP1 then translocates to the mitochondria to mediate its aberrant fission and damage (42). It has been reported that the dengue virus, VSV, SeV, as well as poly(I:C), induce NLRP3 inflammasome activation and are all dependent on the RIP1-RIP3-DRP1 pathway (42, 58). However, reports argue that ROS, brought about by mitochondria damage, is not essential for activation of the NLRP3 inflammasome (59). Instead, mitochondrial membrane potential induced by influenza or EMCV is required for activation of the NLRP3 inflammasome. Under the appropriate mitochondrial membrane potential, NLRP3 will translocate to the mitochondria to combine with mitofusin 2, a mediator of mitochondrial fusion (59).

Accumulation of misfolded protein aggregates is an important activation signal of the NLRP3 inflammasome. A well-known example is that of Alzheimer's disease (AD), which is characterized by the accumulation of amyloid-β peptide (60). The ORF 8b of SARS-CoV forms intracellular aggregates through the valine residue at position 77 (61). This complex acts as the danger signal to induce endoplasmic reticulum stress and lysosomal damage, resulting in NLRP3 inflammasome activation.

Viral infection alters the plasma membrane integrity and ionic efflux, which could lead to programmed cell death and induce the secondary activation of NLRP3 inflammasome. The process of viral replication causes lytic cell death and subsequent potassium efflux, which provides the second signal for NLRP3 inflammasome activation (62). The PB1-F2 protein from IAV induces oxidative stress and the alteration of mitochondrial calcium, leading to apoptosis and NLRP3 inflammasome activation (63).

## Viral Evasion Strategies Targeting The NLRP3 Inflammasome

**Figure 2:**
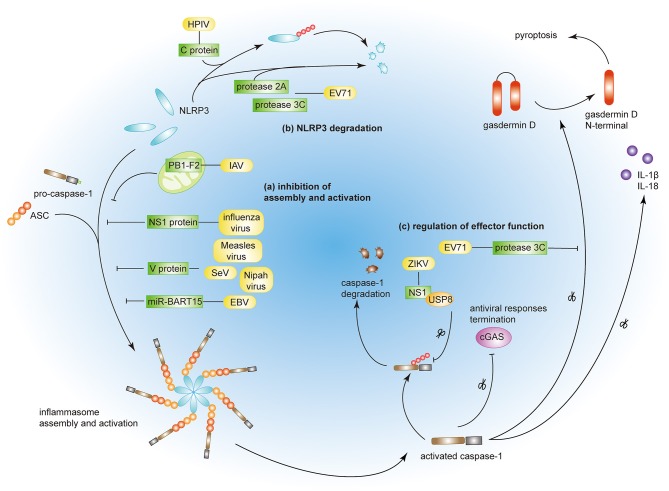
Viral immune evasion strategies by targeting the NLRP3 inflammasome. (a) Influenza virus NS1 protein, measles virus, SeV, and Nipah virus V proteins prevent NRLP3 inflammasome assembly. PB1-F2 of IAV and miR-BART15 of EBV inhibit NLRP3 inflammasome activation. (b) EV71 proteases 2A and 3C and HPIV C protein induce NLRP3 protein degradation. (c) EV71 protease 3C and ZIKV NS1 protein modulate the effector function of the NLRP3 inflammasome by targeting GSDMD and caspase-1, respectively.

Optimal activation of host immunity is crucial for the elimination of invading viruses. However, viruses have evolved strategies to evade immune responses by limiting the activation of the NLRP3 inflammasome. Some viruses have been reported to suppress NLRP3 inflammasome activation to circumvent innate immunity and facilitate viral replication (Figure 2).

Viruses can inhibit both the assembly and the activation of the NLRP3 inflammasome through direct or indirect interactions. Measles virus and paramyxovirus (such as SeV and Nipah virus) V protein, and influenza virus NS1 protein inhibit NLRP3 inflammasome activation by interacting with NLRP3, decreasing the secretion of IL-1β accordingly (64–66). The interaction between viral proteins and NLRP3 prevents the self-oligomerization of NLRP3 as well as the recruitment of ASC, resulting in the block of NLRP3-dependent ASC oligomerization and subsequent inflammasome activation (66). As a regulator of NLRP3 expression, the myeloid-specific microRNA miR-223 downregulates NLRP3 inflammasome activation by binding within the 3′ untranslated region (UTR) of NLRP3 (67). Under these conditions, EBV miR-BART15 specifically targets the miR-223 binding site in the NLRP3 3′-UTR to inhibit NLRP3 activation (68). miR-BART15 can also be transported around non-infected cells through exomes secreted from infected B cells, thereby amplifying this immunosuppressive state. PB1-F2, a viral virulence protein encoded by most IAV strains, could be spliced into different lengths (63). Although PB1-F2 induces excessive NLRP3 inflammasome activation by inducing apoptosis, the mediation of phagolysosome acidification or formation of PB1-F2 aggregates (22, 63), it also could impair NLRP3 inflammasome activation through different mechanism (69). PB1-F2 distributes into the mitochondrial inner membrane space via Tom 40 channels and this mitochondrial location of PB1-F2 attenuates the mitochondrial membrane potential and as a result, inhibits NLRP3 inflammasome activation (69). These conflicting results may be attributed to the different secondary structures of PB1-F2 spliceosomes (70).

NLRP3 ubiquitination and protein degradation is a key regulatory mechanism for NLRP3 inflammasome activation (71, 72). A couple of viral proteins mediate NLRP3 degradation and thus suppress NLRP3 inflammasome activation. The EV71 proteases 2A and 3C directly cleave NLRP3 protein at residues G493-L494 or Q225-G226, respectively (73). HPIV type 3 C protein interacts with NLRP3 and promotes its ubiquitination, thereby mediating NLRP3 proteasomal degradation (31).

A couple of viruses can modulate the effector functions of the NLRP3 inflammasome. The EV71 protease 3C cuts the NLRP3 inflammasome activation effect factor GSDMD, at 193–194 residues, instead of 275–276 residues by caspase-1 (74). This aberrantly cleaved GSDMD product fails to induce cell pyroptosis of the infected cell and, as a result, promote viral replication and attains the objective of viral evasion. Some viruses tend to take the “retreat in order to advance” strategy to maintain their survival. The ZIKV infection is a public health emergency and host IFN-β-associated antiviral innate immunity is essential for the control of this viral infection. Zheng and colleagues discovered that ZIKV infection induces NLRP3 inflammasome activation and deliberately enhances its activation through the NS1 protein (75). NS1 recruits the host deubiquitinase, USP8, to cleave the polyubiquitin chains from caspase-1 so as to inhibit the proteasomal degradation of caspase-1 and, subsequently, amplify the NLRP3 activation signal. However, although the inflammatory response is strengthened by NS1, the large amount of caspase-1 turns out to the cleavage of cGAS, the critical component associated with the antiviral innate immune response. This interplay partly reflects the complex co-evolution between virus and host and may provide potential therapeutics in the future.

## Conclusion And Perspective

Both the NLRP3 inflammasome activation and the subsequent inflammation play significant roles in defending against viral infections. However, aberrant NLRP3 inflammasome activation or chronic inflammation can also lead to severe pathological injury. Accordingly, activation of the NLRP3 inflammasome and its associated inflammation is a double-edged sword for host to defense viral infection. Modulating the NLRP3 inflammasome activity can prove to be a promising strategy for the intervention of viral diseases. In a juvenile mouse model of IAV infection, MCC950, a specific inhibitor of the NLRP3 inflammasome, ameliorates severe NLRP3 inflammasome-mediated lung injury without impairing viral clearance (76).

In this review, we focused on the activation of the NLRP3 inflammasome during viral infection, as well as the immune evasion strategies of viruses. However, the mechanisms of NLRP3 inflammasome activation triggered by viral infection are far from fully elucidated and many questions still remain unanswered. Can NLRP3 sense viral components directly and, if so, how? Are there any receptors that can coordinate with NLRP3 to recognize viruses? Can viral DNA activate NLRP3 through the DNA sensors-RIP1-RIP3 pathway? A virus such as hepatitis B virus (HBV), could not activate NLRP3 inflammasome by itself (77), so how does it escape the surveillance of the immune system? Elucidating the mechanisms of NLRP3 inflammasome activation during viral infection will help us to understand the pathogenesis of inflammation-associated diseases better and discover suitable therapeutic targets for viral diseases.

## Author Contributions

CZ drafted the manuscript and figures. WZ supervised and edited the manuscript and figures.

### Conflict of Interest

The authors declare that the research was conducted in the absence of any commercial or financial relationships that could be construed as a potential conflict of interest.
